# Supplementary material for: Evaluation of Two Adjuvant Formulations for an Inactivated Yellow Fever 17DD Vaccine Candidate in Mice
Source: Vaccines (Basel). 2022 Dec 28;11(1):73. doi: 10.3390/vaccines11010073 (PMC9865672; doi:10.3390/vaccines11010073)
Supplement: Supplementary file 1 [file vaccines-11-00073-s001.zip › vaccines-2038924-supplementary.pdf]

Figure S1: Immunization schedule.

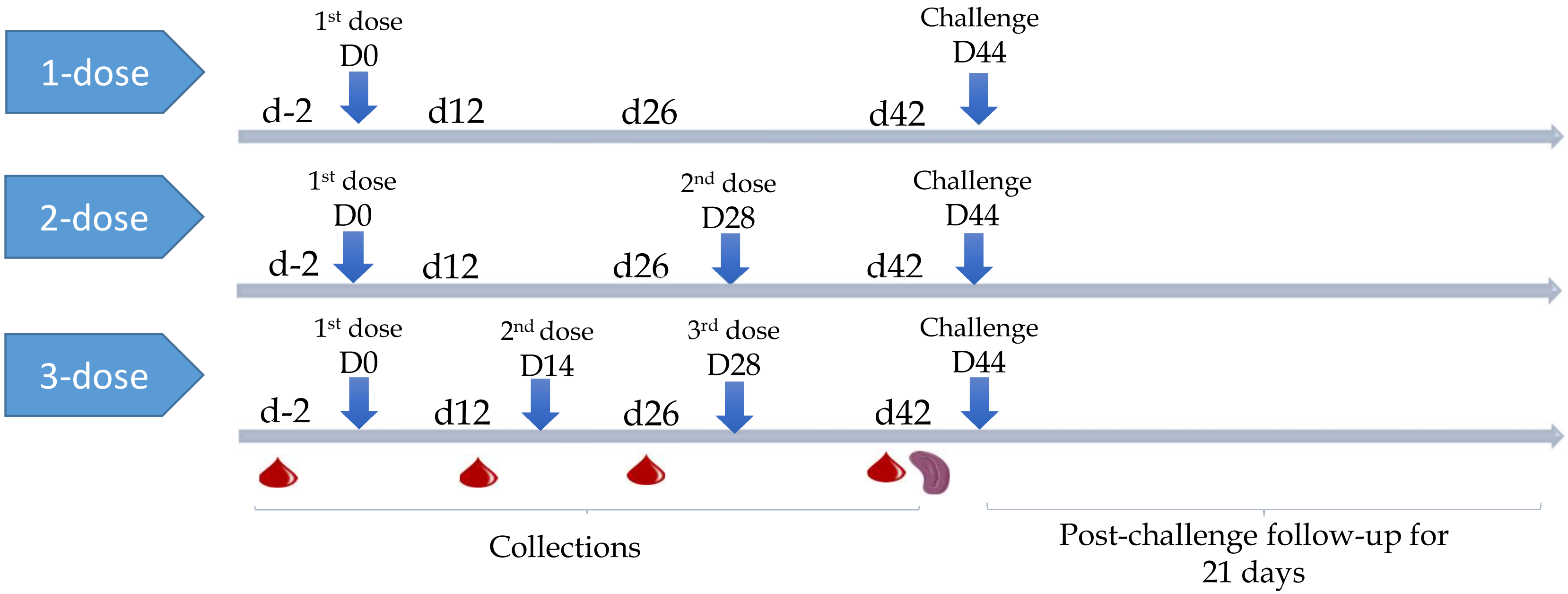

Figure S2: Survival rates after IC challenge of 1st preclinical assay.

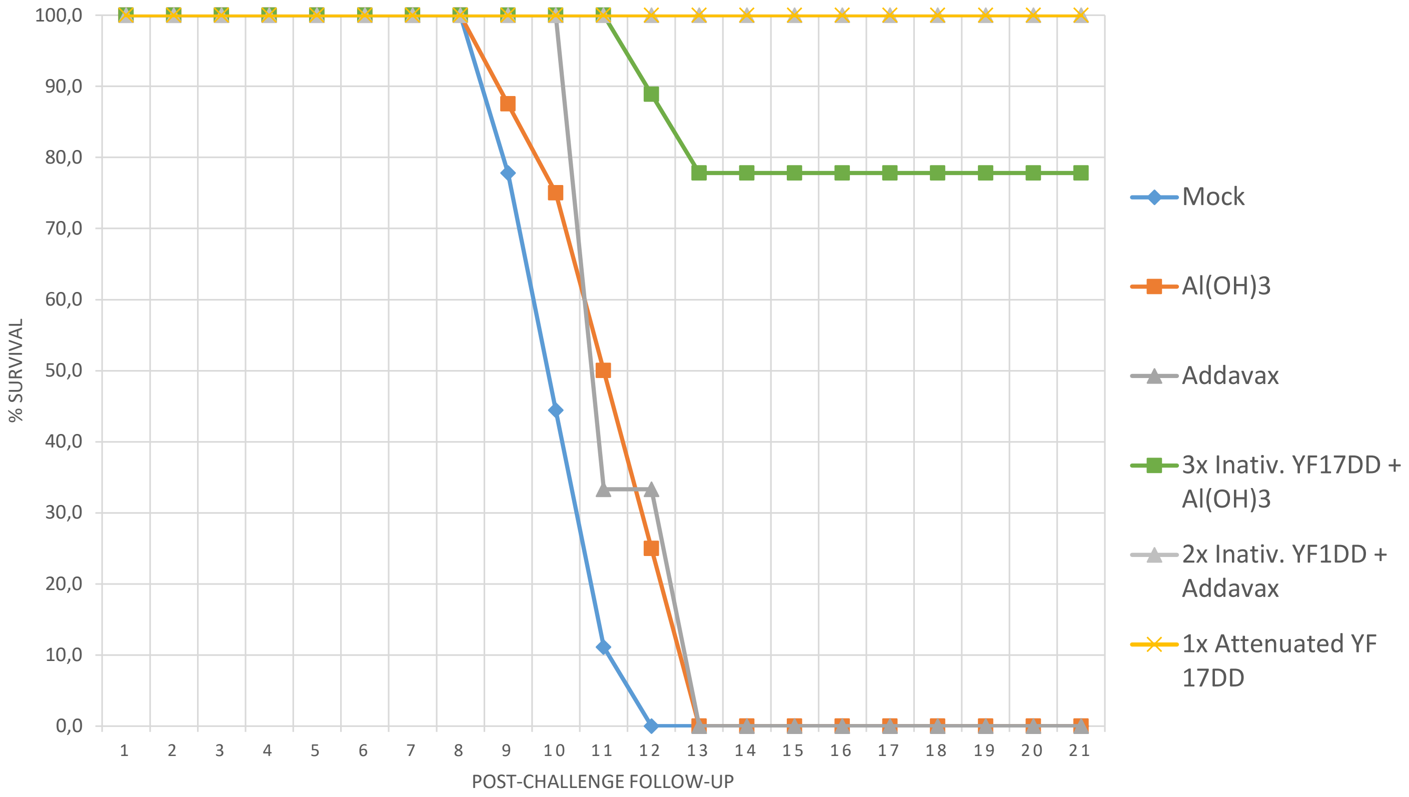

Figure S3: Survival rates after IC challenge of 2nd preclinical assay.

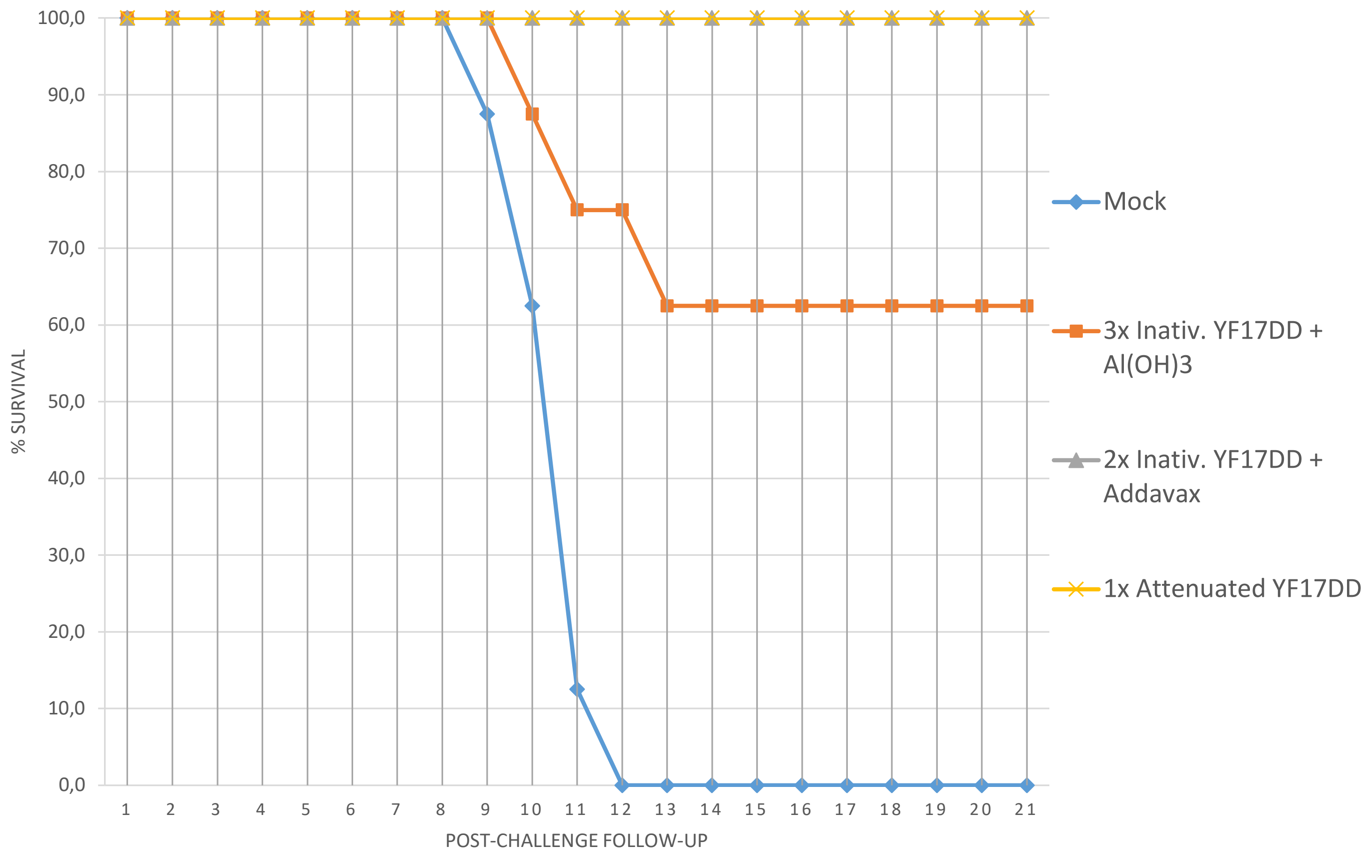

Table S1: Raw data IFN-γ ELISpot of draining lymph node.

	Pre-challenge samples_ IFNY in draining lymph node											
	Mock			3x Inativ. YF/ $Al(OH)_3$			2x Inativ. YF/ AddaVax			Attenuated YF		
**Sample**	**1.9**	**1.10**	**1.11**	**2.9**	**2.10**	**2.11**	**3.9**	**3.10**	**3.11**	**4.9**	**4.10**	**4.11**
**Nº of spots with stimulus**	4	8	6	33	11	5	23	43	35	8	19	13
	2	2	11	40	5	6	14	28	54	11	10	8
**Mean**	**3**	**5**	**8,5**	**36,5**	**8**	**5,5**	**18,5**	**35,5**	**44,5**	**9,5**	**14,5**	**10,5**
**Nº of spots without stimulus**	10	17	28	9	11	5	10	17	28	9	11	5
	16	17	17	7	7	3	16	17	17	7	7	3
**Mean**	**13**	**17**	**22,5**	**8**	**9**	**4**	**13**	**17**	**22,5**	**8**	**9**	**4**
**ConA**	105	118	233	148	97	78	184	272	317	183	182	254
	**SFCs quantification (with peptide stimulus - without stimulus= nº spots/2E+05)**											
	1.9	1.10	1.11	2.9	2.10	2.11	3.9	3.10	3.11	4.9	4.10	4.11
	**-10**	**-12**	**-14**	**28,5**	**-1**	**1,5**	**5,5**	**18,5**	**22**	**1,5**	**5,5**	**6,5**
	**SFCs/ 10^6**											
	1.9	1.10	1.11	2.9	2.10	2.11	3.9	3.10	3.11	4.9	4.10	4.11
	**-50**	**-60**	**-70**	**142,5**	**-5**	**7,5**	**27,5**	**92,5**	**110**	**7,5**	**27,5**	**32,5**

Table S2: Raw data IL-2 ELISpot of draining lymph node.

	Pre-challenge samples_ IL-2 in draining lymph node											
	**Mock**			**3x Inativ. YF/ $Al(OH)_3$**			**2x Inativ. YF/ AddaVax**			**Attenuated YF**		
**Sample**	**1.9**	**1.10**	**1.11**	**2.9**	**2.10**	**2.11**	**3.9**	**3.10**	**3.11**	**4.9**	**4.10**	**4.11**
**Nº of spots with stimulus**	2	4	9	29	8	7	12	30	50	14	8	9
	0	6	5	27	6	5	4	26	37	8	18	10
**Mean**	**1**	**5**	**7**	**28**	**7**	**6**	**8**	**28**	**43,5**	**11**	**13**	**9,5**
**Nº of spots without stimulus**	2	5	4	23	0	0	7	12	27	2	7	4
	0	1	3	2	0	1	2	8	11	2	0	1
**Mean**	**1**	**3**	**3,5**	**12,5**	**0**	**0,5**	**4,5**	**10**	**19**	**2**	**3,5**	**2,5**
**ConA**	271	364	410	424	207	139	432	434	433	281	391	384
	**SFCs quantification (with peptide stimulus - without stimulus= nº spots/2E+05)**											
	1.9	1.10	1.11	2.9	2.10	2.11	3.9	3.10	3.11	4.9	4.10	4.11
	**0**	**2**	**3,5**	**15,5**	**7**	**5,5**	**3,5**	**18**	**24,5**	**9**	**9,5**	**7**
	**SFCs/ 10^6**											
	1.9	1.10	1.11	2.9	2.10	2.11	3.9	3.10	3.11	4.9	4.10	4.11
	**0**	**10**	**17,5**	**77,5**	**35**	**27,5**	**17,5**	**90**	**122,5**	**45**	**47,5**	**35**

Table S3: Raw data IL-4 ELISpot of draining lymph node.

	Pre-challenge samples_ IL-4 in draining lymph node											
	**Mock**			**3x Inativ. YF/ Al(OH)$_3$**			**2x Inativ. YF/ AddaVax**			**Attenuated YF**		
**Sample**	**1.9**	**1.10**	**1.11**	**2.9**	**2.10**	**2.11**	**3.9**	**3.10**	**3.11**	**4.9**	**4.10**	**4.11**
**Nº of spots with stimulus**	1	0	3	1	4	2	1	1	14	4	4	0
	0	3	1	8	1	2	6	10	23	2	5	1
**Mean**	**0,5**	**1,5**	**2**	**4,5**	**2,5**	**2**	**3,5**	**5,5**	**18,5**	**3**	**4,5**	**0,5**
**Nº of spots without stimulus**	1	0	1	15	1	0	2	13	36	1	0	0
	0	0	0	6	0	1	0	2	7	0	0	0
**Mean**	**0,5**	**0**	**0,5**	**10,5**	**0,5**	**0,5**	**1**	**7,5**	**21,5**	**0,5**	**0**	**0**
**ConA**	31	54	87	120	34	16	55	80	100	27	78	57
	**SFCs quantification (with peptide stimulus - without stimulus= nº spots/2E+05)**											
	1.9	1.10	1.11	2.9	2.10	2.11	3.9	3.10	3.11	4.9	4.10	4.11
	**0**	**1,5**	**1,5**	**-6**	**2**	**1,5**	**2,5**	**-2**	**-3**	**2,5**	**4,5**	**0,5**
	**SFCs/ 10^6**											
	1.9	1.10	1.11	2.9	2.10	2.11	3.9	3.10	3.11	4.9	4.10	4.11
	**0**	**7,5**	**7,5**	**-30**	**10**	**7,5**	**12,5**	**-10**	**-15**	**12,5**	**22,5**	**2,5**

Table S4: Raw data IFN-γ ELISpot of brain.

Post-challenge samples_ IFNγ in pool of brain of survivors			
	**3x Inativ. YF/ Al(OH)$_3$**	**2x Inativ. YF/ AddaVax**	**Attenuated YF**
**Nº of spots with stimulus**	916	950	770
	868	833	627
**Mean**	**892**	**891,5**	**698,5**
**Nº of spots without stimulus**	804	747	595
	655	786	555
**Mean**	**729,5**	**766,5**	**575**
**ConA**	1599	1486	1175

SFCs quantification (with peptide stimulus - without stimulus= SFCs/2E+05)

**3x Inativ. YF/ Al(OH)$_3$**	**2x Inativ. YF/ AddaVax**	**Attenuated YF**
**162,5**	**125**	**123,5**

SFCs/ 10^6

**3x Inativ. YF/ Al(OH)$_3$**	**2x Inativ. YF/ AddaVax**	**Attenuated YF**
**812,5**	**625**	**617,5**

Table S5: Raw data IL-2 ELISpot of brain.

Post-challenge samples_ IL2 in pool of brain of survivors			
	3x Inativ. YF/ Al$(OH)_3$	2x Inativ. YF/ AddaVax	Attenuated YF
Nº of spots with stimulus	68	28	41
	33	12	34
**Mean**	**50,5**	**20**	**37,5**
Nº of spots without stimulus	41	8	14
	18	18	7
**Mean**	**29,5**	**13**	**10,5**
**ConA**	300	305	303

SFCs quantification (with peptide stimulus - without stimulus= nº spots/2E+05)

3x Inativ. YF/ Al$(OH)_3$	2x Inativ. YF/ AddaVax	Attenuated YF
**21**	**7**	**27**

SFCs/ 10^6

3x Inativ. YF/ Al$(OH)_3$	2x Inativ. YF/ AddaVax	Attenuated YF
**105**	**35**	**135**

Table S6: Raw data IL-4 ELISpot of brain.

Post-challenge samples_ IL-4 in pool of brain of survivors			
	**3x Inativ. YF/ $Al(OH)_3$**	**2x Inativ. YF/ AddaVax**	**Attenuated YF**
**Nº of spots with stimulus**	51	40	26
	55	34	40
**Mean**	**53**	**37**	**33**
**Nº of spots without stimulus**	5	8	5
	6	7	18
**Mean**	**5,5**	**7,5**	**11,5**
**ConA**	197	221	61

SFCs quantification (with peptide stimulus - without stimulus= nº spots/2E+05)

3x Inativ. YF/ $Al(OH)_3$	2x Inativ. YF/ AddaVax	Attenuated YF
**47,5**	**29,5**	**21,5**

SFCs/ 10^6

3x Inativ. YF/ $Al(OH)_3$	2x Inativ. YF/ AddaVax	Attenuated YF
**237,5**	**147,5**	**107,5**

Table S7: Raw data IgG ELISpot_Memory B cells in draining limph nodes.

	Pre-challenge time_ELISpot IgG memory B cells in draining lymph node											
	**Mock**			**3x Inativ. YF/ $Al(OH)_3$**			**2x Inativ. YF/ AddaVax**			**Attenuated YF**		
**Sample**	**1.9**	**1.10**	**1.11**	**2.9**	**2.10**	**2.11**	**3.9**	**3.10**	**3.11**	**4.9**	**4.10**	**4.11**
**Memory cells IgG spots**	0	11	0	59	13	19	106	122	79	18	32	19
	1	2	0	40	19	12	54	98	75	16	33	16
**Mean**	**0,5**	**6,5**	**0**	**49,5**	**16**	**15,5**	**80**	**110**	**77**	**17**	**32,5**	**17,5**
**Background memory cells**	0	10	0	6	0	2	21	33	24	3	27	23
	**SFCs Quantification (with peptide stimulus - without peptide stimulus= nº of spots/2E+05 cells)**											
	1.9	1.10	1.11	2.9	2.10	2.11	3.9	3.10	3.11	4.9	4.10	4.11
	**0,5**	**-3,5**	**0**	**43,5**	**16**	**13,5**	**59**	**77**	**53**	**14**	**5,5**	**-5,5**
	**SFCs/ 10^6**											
	1.9	1.10	1.11	2.9	2.10	2.11	3.9	3.10	3.11	4.9	4.10	4.11
	**2,5**	**-17,5**	**0**	**217,5**	**80**	**67,5**	**295**	**385**	**265**	**70**	**27,5**	**-27,5**

Table S8: Raw data IgG ELISpot_Plasma cells in brain.

Post- challenge time_ELISpot IgG plasma cells in pool of brain of survivors			
	**3x Inativ. YF/ $Al(OH)_3$**	**2x Inativ. YF/ AddaVax**	**Attenuated YF**
**Plasma cells spots**	64	41	67
	57	50	69
**Mean**	**60,5**	**45,5**	**68**
**Background Cells**	1	10	7
**SFCs Quantification (with peptide stimulus - without peptide stimulus= nº of spots/2E+05 cells)**			
	**3x Inativ. YF/ $Al(OH)_3$**	**2x Inativ. YF/ AddaVax**	**Attenuated YF**
	**59,5**	**35,5**	**61**
**SFCs/ 10^6**			
	**3x Inativ. YF/ $Al(OH)_3$**	**2x Inativ. YF/ AddaVax**	**Attenuated YF**
	**297,5**	**177,5**	**305**

Table S9: Raw data IgG ELISpot_Memory B cells in brain.

**Post- challenge time_ELISpot IgG memory B cells in pool of brain of survivors**			
	**3x Inativ. YF/ $Al(OH)_3$**	**2x Inativ. YF/ AddaVax**	**Attenuated YF**
**Memory cells spots**	13	19	7
	24	39	2
**Mean**	**18,5**	**29**	**4,5**
**Background Cells**	7	16	2
**SFCs Quantification (with peptide stimulus - without peptide stimulus = nº of spots/2E+05 cells)**			
	**3x Inativ. YF/ $Al(OH)_3$**	**2x Inativ. YF/ AddaVax**	**Attenuated YF**
	**11,5**	**13**	**2,5**
**SFCs/ 10^6**			
	**3x Inativ. YF/ $Al(OH)_3$**	**2x Inativ. YF/ AddaVax**	**Attenuated YF**
	**57,5**	**65**	**12,5**
